# Post-transcriptional Gene Regulation in Colitis Associated Cancer

**DOI:** 10.3389/fgene.2019.00585

**Published:** 2019-06-19

**Authors:** Gang Chen, Yuan Feng, Xuezheng Li, Zhe Jiang, Bei Bei, Lin Zhang, Yueqing Han, Yanwu Li, Ning Li

**Affiliations:** ^1^School of Traditional Chinese Materia Medica, Shenyang Pharmaceutical University, Shenyang, China; ^2^Beijing Key Laboratory of Bio-characteristic Profiling for Evaluation of Rational Drug Use, Beijing Shijitan Hospital, Capital Medical University, Beijing, China; ^3^Department of Pharmacy, Yanbian University Hospital, Yanji, China; ^4^Pi-Wei Institute, Guangzhou University of Chinese Medicine, Guangzhou, China

**Keywords:** colitis-associated cancer, pedunculoside, miR-31-5p, miR-223-3p, let-7f-5p

## Abstract

Colitis-associated cancer (CAC) has been linked to microRNA (miRNA) aberrant expression elicited by inflammation. In this study, we used the AOM/DSS-induced CAC mice model to explore the ectopic expression of miRNAs in the precancerous stage of CAC. As a result, we found that miR-31-5p, miR-223-3p, and let-7f-5p were dysregulated during the development of intestinal dysplasia. Subsequently, we first identified the role of these three miRNAs in CAC. Adenomatous polyposis coli (APC) was revealed as a new target of miR-223-3p, and solute carrier family 9- subfamily A-member 9 (SLC9A9) and APC membrane recruitment protein 3 (AMER3) were suggested as two new targets for let-7f-5p. For miR-31-5p, we proved that it can target LATS2 mRNA so as to modulate Hippo pathway in Caco2 cells. Second, to examine if targeting these three miRNAs would lead to CAC prevention, pedunculoside, a natural triterpene glycoside capable of rescuing the down-regulation of LATS2 and APC caused by either miR-31-5p or miR-223-3p overexpression, respectively, was used in the *in vivo* AOM/DSS-induced CAC model. The results showed that pedunculoside (25 mg/kg) substantially mitigated the damage to mice intestine caused by DSS/AOM. These results suggested that miRNAs-elicited post-transcriptional regulation is involved in the pathogenesis of CAC, and CAC can be prevented through targeting key miRNAs that are ectopically expressed in CAC.

## Introduction

The first case of inflammatory bowel disease (IBD) linked to colorectal cancer (CRC) was reported by Crohn and Rosenberg (1925), and from then on, plenty of efforts has been made to substantiate this potential association between IBD and CRC. Nowadays IBD is deemed as an important risk factor for the development of colon cancer, namely colitis-associated cancer (CAC), which refers to the type of colon cancer preceded by detectable IBD in clinic, such as Crohn’s disease (CD) or Ulcerative colitis (UC). UC increases cumulative risk of CAC by up to 18–20%, and for CD the number is up to 8%, after 30 years of disease (Eaden et al., 2001; Canavan et al., 2006; Rubin et al., 2012). However, so far the mechanism of inflammation-associated colon cancer is still elusive.

To facilitate the understanding of the underlying mechanism in CAC, mouse model has been extensively adopted by using AOM/DSS to mimic the presentation of oncogenic and inflammatory factors in CAC (Park et al., 2017; Chung et al., 2018; Guo et al., 2018). In this model, AOM is a chemical carcinogen functions through DNA alkylation. After injection, AOM is metabolized in the liver, followed by the excretion in the bile. And its carcinogenicity can be reinforced by further bacterial flora-driven metabolism. In the same time, DSS is included in the drinking water to induce colitis. First, AOM injection followed by one cycle of DSS is used in this model. While, to further simulate the chronic inflammatory states, three cycles of DSS is often utilized after the AOM injection so as to induce a chronic colitis. In this model, tumor formation is accelerated, and therefore this AOM/DSS-induced CAC model is also usually used for assessing the CAC preventive drug efficiency.

MicroRNAs (miRNAs) are non-coding RNAs that are ∼21nt, being capable of regulating gene expression post-transcriptionally. Interestingly, miRNAs are linked to the pathogenesis of both IBD and CRC (Li et al., 2017; Tili et al., 2017; Soroosh et al., 2018). However, the marked disparity among different reports is one of the confusing problem while exploring the miRNA-based mechanism for CAC. And this disparity was basically attributed to the difference of course and/or stage of CAC. In this line of thinking, as opposed to cancerous stage in which solid tumors occur, we are interested in exploring ectopic gene expression in the precancerous stage of CAC, and thereby discover novel therapeutic targets for the screening of CAC preventive natural drugs.

Thus, in this study, we used AOM/DSS-induced CAC model to explore the ectopic expression of miRNAs in the precancerous stage of CAC, and predicted their direct targets so as to clarify the post-transcriptional gene regulation in the CAC pathogenesis. Moreover, through constructing the *in vitro* model simulating the *in vivo* miRNA expression patterns, one natural small molecular pedunculoside that can target the *in vivo* aberrantly expressed miRNAs was found, and was subjected to the AOM/DSS-induced CAC experiment to further confirm the effect of miRNAs in CAC.

## Materials and Methods

### Cell Culture, Animal, and Reagents

Human epithelial colorectal adenocarcinoma cell line Caco-2 was purchased from Kunming Cell Bank. Cells were cultured in MEM-medium (for Caco-2 cells), supplemented with 10% fetal bovine serum (FBS), 100 U/ml penicillin and 100 μg/ml streptomycin (all available from Invitrogen, Grand Island, NY, United States). All cultures were maintained in a humidified environment with 5% CO_2_ at 37°C.

Male or female C57BL/6 mice (12 weeks of age) were obtained from Guangdong experimental animal research center (certification code: SCXK (YUE) 2013-0002). All experiments were performed in the specific pathogen free unit of the Guangzhou University of Chinese Medicine (Animal experimental registration code: SYXK (YUE) 2013-0001). The study protocol was approved by the Ethics Committee for Animal Experiments of Guangzhou University of Chinese Medicine and all efforts were made to minimize suffering. Azoxymethane (AOM) (sigma, Cat. #A5486-25MG) was reconstituted to a final concentration of 10 μg/μL when used. Dextran sulfate sodium (DSS), 36–50 kDa (MP Biomedicals, cat. #160110-100G) was prepared to 2.5% DSS by dissolving 2.5 g DSS per 100 ml dH_2_O when needed and changed every day for DSS cycle in experiment.

### MicroRNA Array

MicroRNA array: RNA of sufficient quality from 2 group samples (*n* = 2, total 4 samples) was submitted to KangChen-Biotech (Shanghai, China). RNA groups above mentioned by applying the miRCURY locked nucleic acid (LNA) microarray platform (Exiqon, Denmark). All procedures were carried out according to manufacturer’s protocol. Gene Pix 4000B scanner and GenePix Pro 6.0 software (Axon Instruments, Union City, CA, United States) were used to acquire images. Background subtraction and normalization were performed. Two-fold or larger change was set as a threshold of significant difference.

### Transfection With miRNAs

Pre-miR-31, Pre-miR-223, and Pre-let-7f were purchased from Ambion (Austin, TX, United States). Untreated Caco2 cells, growing exponentially, were plated at 2 × 10^7^/well in 2.5 ml DMEM medium for 24 h on six-well plates. Once cells reached about 50% confluence, transfection was conducted. Lipo2000 from Invitrogen (Carlsbad, CA, United States) was used in all transfection processes according to the manufacturer’s instructions.

### Preparation for Pedunculoside

The whole plant of *Ilex rotunda* was purchased from Guangzhou in 2016. Dr. Gao Feng authenticated the plant, and a voucher specimen was deposited in Shenyang Pharmaceutical University (Reference No. 2016-0105). The detailed process of isolation and identification for pedunculoside was described in the Supplementary Data.

### Western Blot Analysis

Whole-cell lysates were prepared using 2% SDS, sonicated and centrifuged (15000 *g*) at 4°C for 15 min. The supernatants were boiled for 5 min and size-fractionated by SDS/PAGE (10% acrylamide). After transferring proteins on to nitrocellulose filters, the blots were incubated with primary antibodies recognizing APC, AMER3, SLC9A9, GPADH, β-actin, and factor in the Hippo pathway (Cell Signaling Technology, Beverly, MA, United States); following incubation with secondary antibodies, immunocomplexes were developed by using chemiluminescence.

### Reverse Transcription (RT) and Quantitative Real-Time PCR Analyses

Quantitative real-time PCR was performed using SYBR Premix EXtagII (TaKaRa, Dalian, China) in the PRISM 7900HT system (Applied Biosystems, Carlsbad, CA, United States). For miRNA quantification: Bulge-loop miRNA qRT-PCR Primer Sets (one RT primer and a pair of qPCR primers for each set) specific for miR-31-5p, miR-223-3p, and let-7f-5p were designed by RiboBio (Guangzhou, China). U6 small nuclear RNA (snRNA) was used as endogenous control. Total RNA was isolated from cells using an RNeasy Mini Kit (Qiagen, Valencia, CA, United States). RT-PCR for mRNAs were performed using ABI TaqMan Gene Expression Assays (Applied Biosystems, Foster City, CA, United States). RNA was reverse transcribed by using the high-capacity cDNA archive kit (Applied Biosystems). RT-PCR and subsequent calculations were performed by the Step One Plus Real-time PCR system (Applied Biosystems), which detected the signal emitted from fluorogenic probes during PCR. All samples were analyzed three times. The qRT-PCR results were expressed relative to GAPDH expression levels at the threshold cycle (Ct), which were then converted to fold change (2^-ΔΔCt^).

### Luciferase Reporter Assay

The human SLC9A9, APC, and AMER3 3′ untranslated region (UTR) fragment containing putative binding sites for miR-223-3p or let-7f-5p were amplified by PCR from human genomic DNA. The mutant 3′-UTRs were obtained by overlap extension PCR. The fragments were cloned into a pmirGLO reporter vector (Promega, Madison, WI, United States), downstream of the luciferase gene, to generate the recombinant vectors pmirGLO-WT and pmirGLO-MUT. For the luciferase reporter assay, Caco2 cells were co-transfected with miRNA (pre-miR or scrambled-miR negative control) and reporter vectors (pmirGLO-WT reporter vectors or pmirGLO- MUT reporter vectors), using lipo2000. Luciferase activities were measured with a Dual-Luciferase assay kit (Promega, Madison, WI, United States) according to manufacturer’s instructions at 24 h post-transfection. Experiments were repeated three times in triplicate.

### Animal Experiments

Weigh mice (*n* = 15 for each group) and inject AOM (12.5 mg/kg) i.p. using a 1 ml sterile syringe. After 1 week 2.5% DSS was prepared to drink for 5 days, followed by 2 weeks of regular water and normal feed. Then start a second cycle of 2.5% DSS for 5 days followed by 2 weeks of recovery. At the same time, peduncloside (20 mg/kg, p.o. per day) treatment was started at the fourth week and continued for 5 weeks. Mice were killed 2 weeks after the end of the last round of DSS by cervical dislocation, the whole colon was removed and split in two along the central axis for measurement and histopathological staining. And the colon tissues were stored at -80°C for RNA and protein expression analysis.

### Immunohistochemistry

We used paraffin-embedded sections of colonic samples from normal, CAC model and peduncloside treatment mice for the immunohistochemistry analysis. Phospho-YAP (Ser127) (CST #13008), YAP/TAZ (CST#8418), APC (ARG52491) antibodies were used according to manufacturer’s instructions and incubated overnight at 4°C. DAB kit and Hematoxylin nuclear counterstain were used for staining as the instructions.

### Statistics

Results are expressed as mean ± S.D. in experiment. Statistically significant values were compared by using one-way ANOVA analysis of Prism 6, and *P* < 0.05 indicated statistical significance.

## Results

### MiR-31-5p, MiR-223-3p, and Let-7f-5p Were Dysregulated in CAC Mice Model

**FIGURE 1:**
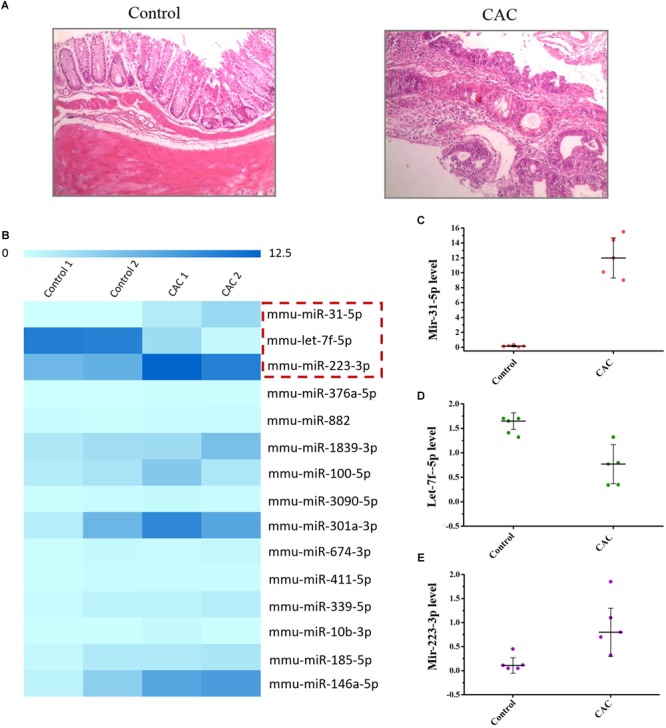
The miRNA array of 8 week CAC model of C57BL/6 mice. **(A)** The microscopic results (H/E) results for the control and CAC model group (*n* = 10). **(B)** The miRNA array results for the control and CAC model group (*n* = 2). **(C–E)** RT-qPCR results for miR-31-5p, miR-223-3p, and let-7f-5p (*n* = 5).

C57BL/6 mice were used to construct the CAC model induced by AOM/DSS method. The microscopic results (H/E) showed that severe intestinal dysplasia, even mucosa neoplasia, could be observed for the mice in the model group at 8 weeks, while mice in the control group bear healthy intestine without any atypical hyperplasia (Figure 1A), suggesting that mice in the 8-week model group are at the precancerous stage. For we were focused on the exploration of the miRNA-based mechanism for CAC prevention, which means we were inclined to determine the stage right before the tumor initiation, and therefore this 8-week CAC model of severe atypical hyperplasia perfectly fell into the scope of our interests. Moreover, the miRNA array was performed to examine the dysregulated miRNAs in mice at 8 weeks. Comparing to those in the control group, mice in the model group showed much higher levels of miR-31-5p and miR-223-3p, but a lower level of let-7f-5p (Figure 1B). Subsequently the expression of these miRNAs were further confirmed by RT-qPCR method, and the result of which proved the up-regulated miR-31-5p and miR-223-3p expression, along with the attenuated let-7f-5p level in the mice of the CAC model group (Figure 1C–E). Thus, miR-31-5p, miR-223-3p, and let-7f-5p were considered to be associated with the oncogenesis for this 8-week CAC model.

### Adenomatous Polyposis Coli (APC) mRNA Is Targeted by MiR-223-3p

**FIGURE 2:**
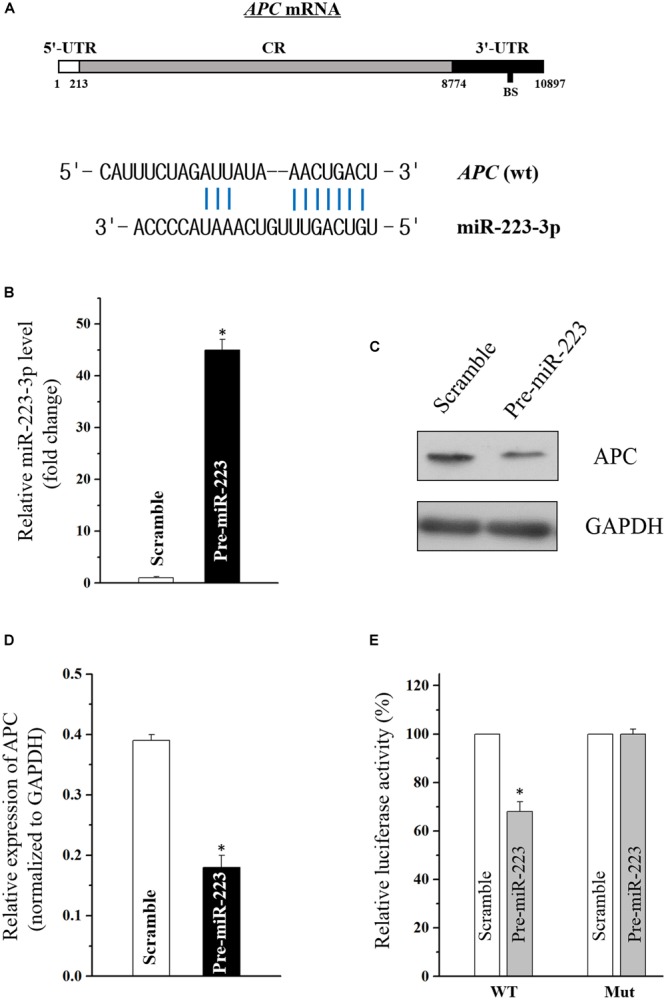
**(A)** Schematic representations of the APC mRNA depicting predicted the binding site (BS) for miR-223-3p in its 3′-UTR. **(B)** Levels of miR-223-3p 48 h after transfection with pre-miR-223 or control scramble. Values are means ± S.E.M. from three separate experiments. ^∗^*P* < 0.05 compared with cells transfected with control scramble. **(C)** Representative immunoblot of APC in cells described in **(B)**. **(D)** Levels of APC mRNA 48 h after transfection with pre-miR-223 or control scramble. Values are means ± S.E.M. from three separate experiments. ^∗^*P* < 0.05 compared with cells transfected with control scramble. **(E)** Overexpression of miR-223-3p significantly inhibited the WT but not the Mut luciferase activity of APC in Caco2 cells. ^∗^*P* < 0.05 compared with cells transfected with control scramble.

Using the standard online software (TargetScan and RNA22), we found that there are predicted binding sites of miR-223-3p within the 3′-UTR of APC (adenomatous polyposis coli) mRNA (Figure 2A), suggesting that miR-31-5p directly interacts with the APC mRNA so as to regulate its expression. To examine the effect of miR-223-3p on APC expression, miR-223-3p was overexpressed by transfection with the miR-223 precursor (pre-miR-223) in Caco2 cells. As shown in Figure 2B, transfection of pre-miR-223 increased the miR-223-3p level remarkably and decreased the APC protein level (Figure 2C,D). Furthermore, luciferase reporter assays were conducted and revealed that miR-31-5p inhibited WT, but not the Mut luciferase activity of APC in Caco2 cells (Figure 2E). Taken together, these results appear to indicate that APC is a direct target gene of miR-223-3p in Caco2 cells.

### Solute Carrier Family 9-Subfamily A-Member 9 (SLC9A9) and APC Membrane Recruitment Protein 3 (AMER3) Are Novel Targets of Let-7f-5p

**FIGURE 3:**
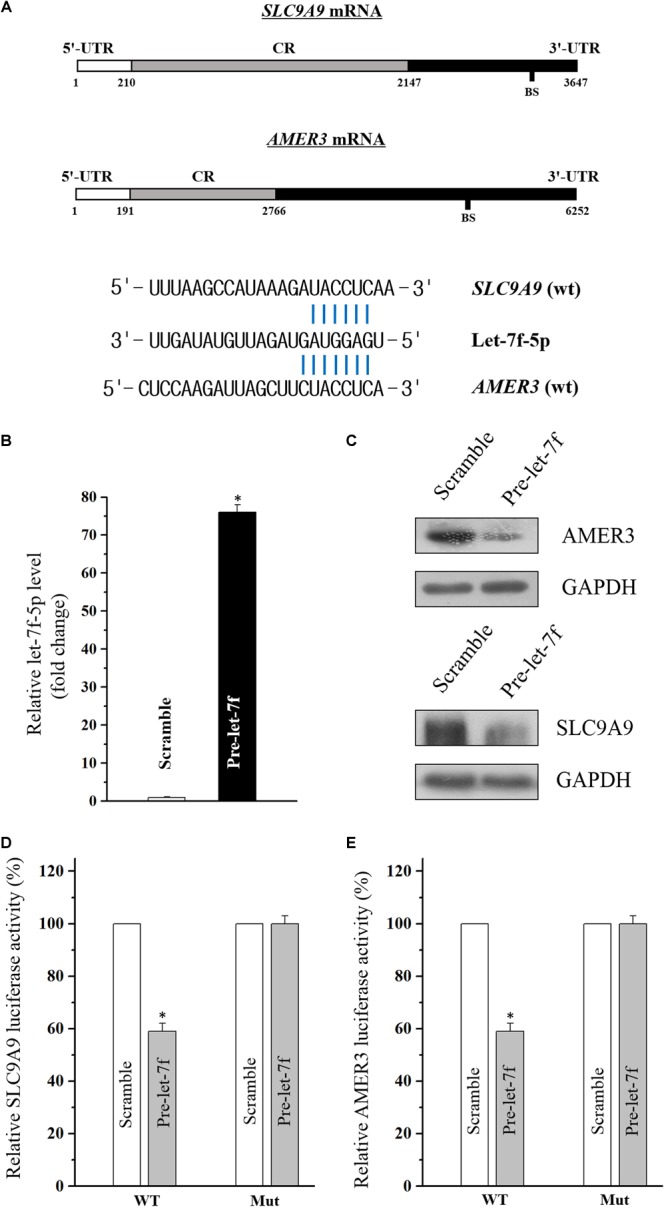
**(A)** Schematic representations of the AMER3 and SLC9A9 mRNAs depicting predicted binding sites (BS) for let-7f-5p in their 3′-UTR. **(B)** Levels of let-7f-5p 48 h after transfection with pre-let-7f or control scramble. Values are means ± S.E.M. from three separate experiments. ^∗^*P* < 0.05 compared with cells transfected with control scramble. **(C)** Representative immunoblots of AMER3 and SLC9A9 in cells described in **(B)**. **(D,E)** Overexpression of let-7f-5p significantly inhibited the WT but not the Mut luciferase activities of AMER3 and SLC9A9 in Caco2 cells. ^∗^*P* < 0.05 compared with cells transfected with control scramble.

Through the target analysis via TargetScan, predicted binding sites of let-7f-5p within the 3′-UTR of the SLC9A9 and AMER3 mRNAs were found (Figure 3A), indicating let-7f-5p could suppress SLC9A9 and AMER3 expression post-transcriptionally by directly interacting with SLC9A9 and AMER3 mRNAs. To verify this predicted regulatory effect of let-7f-5p, transfection experiment of let-7f-5p precursor (pre-let-7f) was conducted to increase its level. Transfection of let-7f-5p precursor significantly increased the let-7f-5p level, and the overexpressed let-7f-5p potently attenuated SLC9A9 and AMER3 expression (Figure 3B,C). Moreover, luciferase reporter assays revealed that let-7f-5p inhibited WT, but not the Mut luciferase activity of SLC9AG and AMER3 in Caco2 cells (Figure 3D,E). On the whole, these results suggested that SLC9AG and AMER3 are direct target genes of let-7f-5p in Caco2 cells.

### MiR-31-5p Suppresses Large Tumor Suppressor Kinase 2 (LATS2) so as to Suppress Hippo Pathway in Caco2 Cells

**FIGURE 4:**
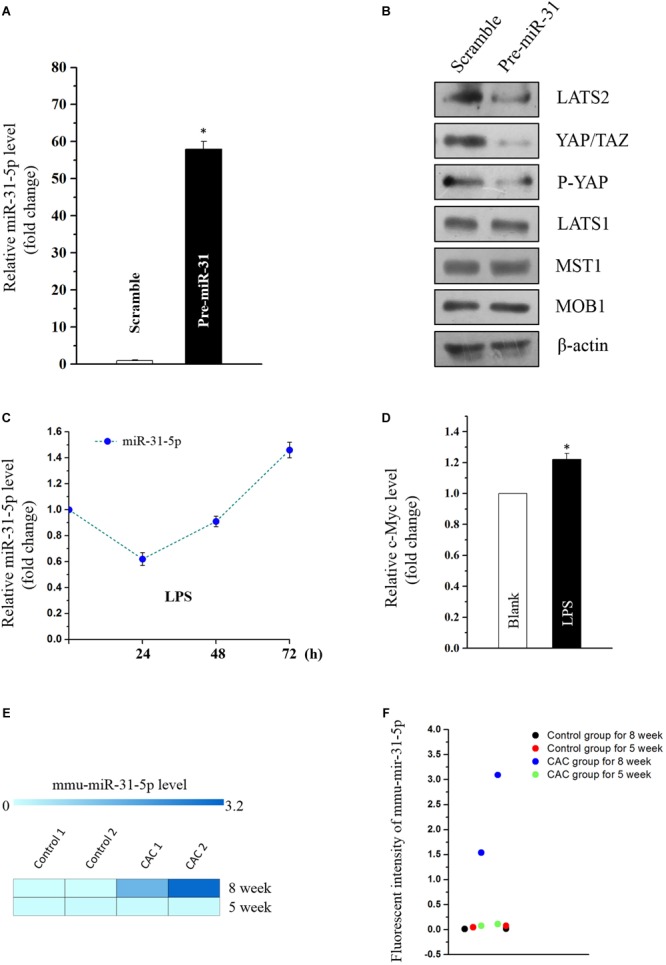
**(A)** Levels of miR-31-5p 48 h after transfection with pre-miR-31 or control scramble. Values are means ± S.E.M. from three separate experiments. ^∗^*P* < 0.05 compared with cells transfected with control scramble. **(B)** Representative immunoblots of factors in Hippo pathway in cells described in **(A)**. **(C)** Changes in the level of miR-31-5p as measured by quantitative PCR analysis in Caco2 cells treated with LPS (2 μg/mL) for 0, 24, 48, and 72 h. Values are means ± S.E.M. from three separate experiments. **(D)** Changes in the level of c-MYC as measured by quantitative PCR analysis in Caco2 cells treated with LPS (2 μg/mL) for 72 h. Values are means ± S.E.M. from three separate experiments. ^∗^*P* < 0.05 compared with the blank group. **(E,F)** Fluorescent intensities of miRNA assay for miR-31-5p in 5 and 8 week CAC models.

Emerging evidence indicates that miR-31 plays a dual role in tumorigenicity. However, whether miR-31-5p plays as an oncogene in CAC and its potential target molecules are still unclear. Aforementioned results of miR-31-5p, whose basal level in CAC group was almost a hundred times as high as in the control group (Figure 1B,C), indicated the potential importance of miR-31-5p in CAC. Furthermore, we found miR-31-5p could modulate Hippo pathway in Caco2 cells via repressing LATS2 although miR-31 was reported to target LATS2 in other cancers (Gao et al., 2017; Luan et al., 2017). First, miR-31-5p was overexpressed by transfection of its precursor (pre-miR-31) (Figure 4A). Western blot analysis showed that LATS2 was significantly decreased by miR-31-5p overexpression (Figure 4B). Second, to investigate if miR-31-5p could affect the up-stream genes of LATS2 so as to decrease LATS2 protein level, MST1 and MOB1 were examined through western blot analysis after pre-miR-31 transfection. The results revealed that miR-31-5p specifically targeted LATS2 without affecting LATST1, MST1 and MOB1 expression (Figure 4B). Subsequently, we found that the decrease of LATS2 by miR-31-5p definitely affected the down-stream genes of LATS2, such as p-YAP and YAP/TAZ (Figure 4B). Thus, these evidence indicated that in Caco2 cells, miR-31-5p modulates the Hippo pathway by specifically targeting LATS2.

MiR-31 was reported to be overexpressed in UC, and was deemed as one of the prognostic biomarkers for stage II and III colon cancer (Gao et al., 2017; Jacob et al., 2017; Luan et al., 2017). This prompted us to investigate if inflammation-induced factors would cause the overexpression of miR-31-5p so as to exacerbate the tumorigenesis of colon cancer. Thus, miR-31-5p and its down-stream gene c-MYC were evaluated in the LPS-treated Caco2 cells. The results showed that only long-term treatment of LPS (72 h) could lead to miR-31-5p overexpression (Figure 4C). And the down-stream gene c-Myc of miR-31-5p was also up-regulated by 72-h treatment of LPS in Caco2 cells as expected (Figure 4D). Interestingly, similar result was observed in the miRNA array for the 5-week CAC model, in which comparing to the 8-week CAC model, miR-31-5p was basically not up-regulated (Figure 4E,F). All these results suggested that long-term inflammation in intestinal cells may contribute to the aberrant expression of miR-31-5p and thereby its down-stream oncogenic factors.

### Pedunculoside Reverses the Suppression of LATS2 and APC in a Caco2 Cell Model in Which MiR-31-5p, MiR-223-3p, and Let-7f-5p Were Manipulated in Accordance With the Result of the miRNA Array

**FIGURE 5:**
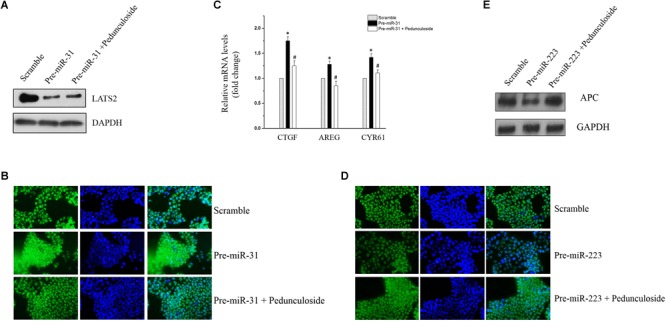
**(A)** Representative immunoblot of pedunculoside (10 μM) restoring LATS2 levels that were down-regulated by miR-31-5p overexpression. **(B)** Representative immunofluorescent staining results of pedunculoside (10 μM) restoring the YAP/TAZ level after miR-31-5p overexpression. **(C)** RT-qPCR results of pedunculoside (10 μM) decreasing CTGF, AREG, and CYR61 levels that were increased by transfection of miR-31 precursor. Values are means ± S.E.M. from three separate experiments. ^∗^*P* < 0.05 compared with the scramble group, and ^#^*P* < 0.05 compared with the Pre-miR-31 group. **(D)** Immunofluorescent staining results of pedunculoside (10 μM) restoring the APC protein level. **(E)** Representative immunoblot of pedunculoside (10 μM) restoring the APC protein level.

To construct an *in vitro* model for the screening of chemopreventive agents against CAC, Caco2 cells were transfected with precursors (for miR-31-5p and miR-223-3p) or the siRNA (for let-7f-5p) to simulate the aberrant expression pattern of miRNAs in the aforementioned CAC mice model. To be noted, western blot analysis showed that pedunculoside, a natural ursane type of triterpenoid, reversed the decreasing tendency of LATS2 caused by miR-31-5p overexpression (Figure 5A). Immunofluorescent staining substantiated that pedunculoside could reverse the miR-31-5p-induced increase of YAP/TAZ (Figure 5B), which is the down-stream gene of LATS2, and therefore abrogated the mRNA overexpression of oncogenic genes caused by miR-31-5p overexpression, such as CTGF, AREG and CYR61 that are down-stream genes of LATS2/YAP/TAZ in the Hippo pathway (Figure 5C). The similar outcome was also found in miR-223-3p overexpression, and the results of which showed that pedunculoside could restore APC protein level lowered by miR-223-3p overexpression (Figure 5D,E). Although pedunculoside did not reverse the effect of let-7f-5p on AMER3 and SLC9A9 (data not shown), this “miR-31-5p and miR-223-3p counteractive” feature of pedunculoside still makes it a good candidate for CAC prevention.

### Pedunculoside Prevents CAC in AOM/DSS-Induced CAC Mice

**FIGURE 6:**
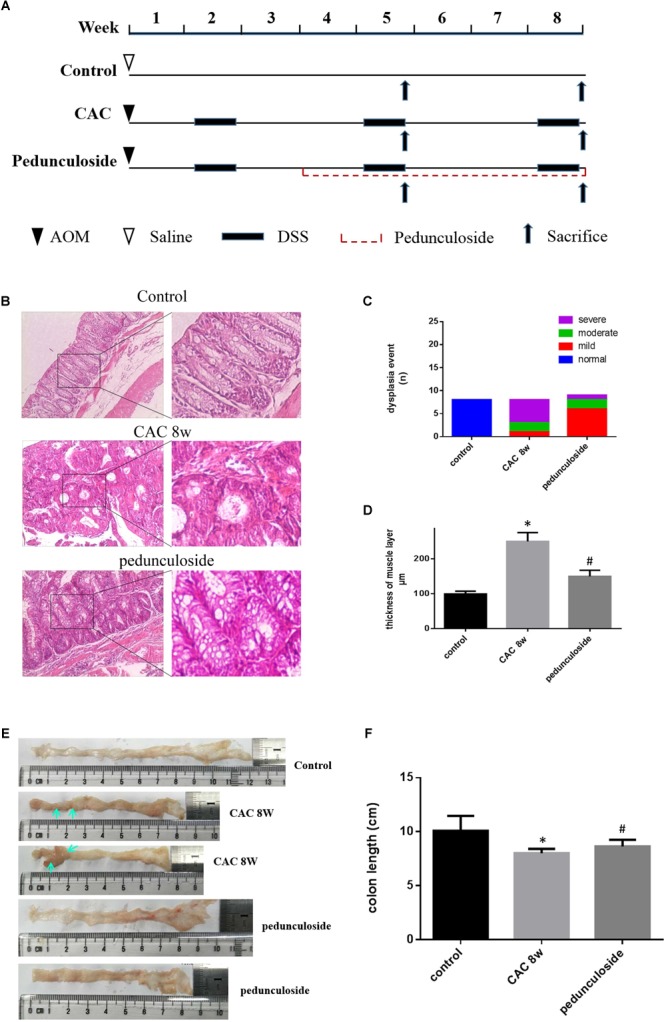
**(A)** Schematic overview of pedunculoside administration. Colons were removed at week 5 and 8 after the mice were administered pedunculoside or a vehicle control between week 2 and 8 (*n* = 15 for each group). **(B)** Examples of HE stained sections representing normal colon image in control mice (top), severe dysplasia (or colon carcinoma) in CAC mice (middle) and moderate dysplasia in treated mice with pedunculoside (bottom). **(C)** Colonic epithelial dysplasia event showed in different group. The degree of dysplasia was characterized as severe, moderate, and mild according to the distribution and extent of epithelial dysplasia (mild dysplasia: slight dysplasia in the lower 1/3 of the epithelium; moderate: dysplasia in the lower 2/3 of the epithelium; high-grade dysplasia in the areas larger than 2/3 of the epithelium). **(D)** Thickness of muscle layer was evaluated at microscope (100×). Three slices, five spots each, were used for the assessment of muscle layer by Pathology Image Analysis System. ^∗^*P* < 0.05 compared with control group; ^#^*P* < 0.05 compared with CAC 8 week group. **(E)** Representative images of colon in normal mice and colorectal tumor (yellow arrow) in CAC mice, thickness and mucosal hyperemia in treatment mice (pedunculoside). **(F)** Colon length of every mice was detected and compared in different group. ^∗^*P* < 0.05 compared with control group; ^#^*P* < 0.05 compared with CAC 8 week group.

**FIGURE 7:**
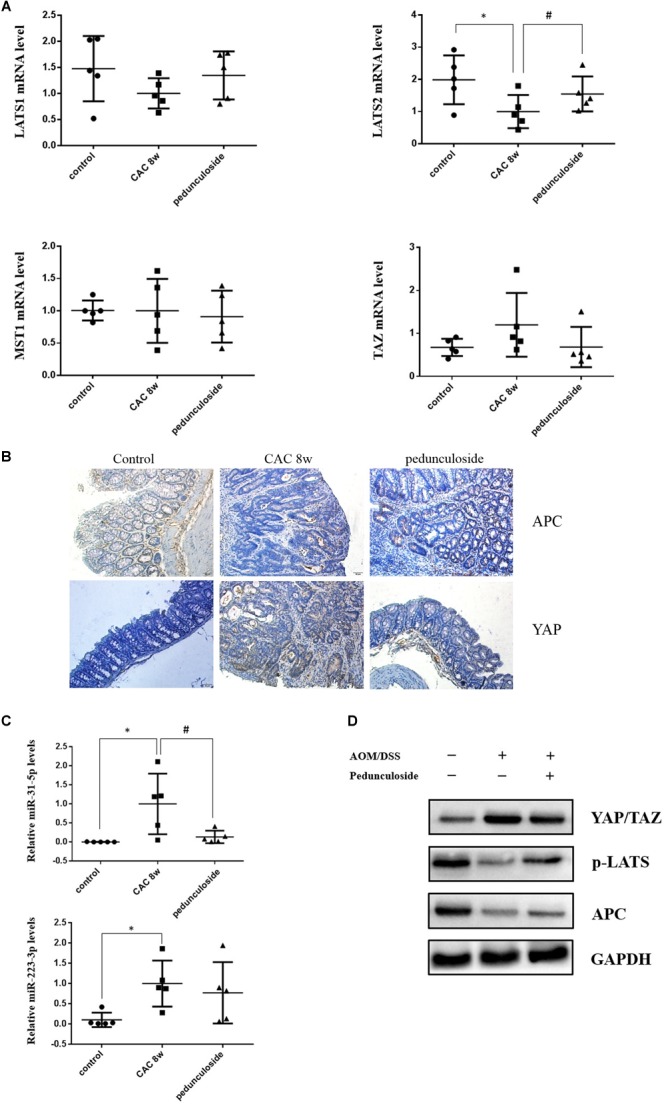
**(A)** Relative mRNA levels of Lats1, Lats2, TAZ, and MST1 in different group mice colon sample, based on real-time PCR (*n* = 5). ^∗^*P* < 0.05 compared with control group; ^#^*P* < 0.05 compared with CAC 8 week group. **(B)** Representative immunostaining for APC and YAP in colon paraffin section (×100). Top left: APC positive staining was mainly distributed in cytoplasm of normal section of colon (brown color); Top middle: In the CAC group, APC positive staining attenuated in the high-grade dysplasia regions (brown color); Top right: APC positive staining increased in the pedunculoside-treated CAC mice (brown color). Bottom left: The YAP level is low in normal group (brown color); Bottom middle: YAP positive staining increased in the nucleus for CAC mice (brown color); Bottom right: YAP positive staining attenuated in the pedunculoside-treated CAC mice (brown color). **(C)** Real-time PCR data showed miR-31-5p and miR-223-3p levels increased obviously in CAC model group, while miR-31-5p expression decreased after pedunculoside administration (*n* = 5). ^∗^*P* < 0.05 compared with control group; ^#^*P* < 0.05 compared with CAC 8 week group. **(D)** Western blot analysis for YAP/TAZ, p-LATS, and APC in different group colon samples. GAPDH was used as loading control (*n* = 5).

To further substantiate the screening results in the aforementioned Caco2 cell model, the *in vivo* experiment was performed using the same aforementioned CAC model (Figure 6A). Histological assessment (HE, 100×) showed that in the CAC group the normal structure of glands disappeared with enlarged nucleus, and in the pedunculoside treatment group glands were arranged in disorder with a reduced number of enlarged nucleus (Figure 6B). The event summarization for the dysplasia suggested the different degree of colonic dysplasia: 5 severe, 2 moderate and 1 mild colonic mucosa dysplasia were observed in CAC model group, while 1 severe, 2 moderate and 6 mild dysplasia appeared in pedunculoside treatment group (Figure 6C). As shown in Figure 6D, the CAC model group had an increased thickness of colonic muscle layer compared with control group (99.67 ± 3.24 μm vs. 250.7 ± 10.31 μm, *P* < 0.01). However, treatment with pedunculoside alleviated the thickened muscle layer caused by AOM/DSS (150.20 ± 7.16 μm, *P* < 0.01). Moreover, the abnormal morphology showed colonic mucosa hyperemia and even tumor formation (*in situ* carcinoma) in CAC model, whereas no *in situ* carcinoma were observed in the mice treated with pedunculoside for 5 week (from 4 to 8 week) as shown in Figure 6E. The colon length of CAC mice was significantly shorter than that of the control group (10.1 ± 1.37 cm vs. 8.02 ± 0.40 cm, *P* < 0.05) (Figure 6F). In contrast, pedunculoside treatment (20 mg/kg) could prevented the shortening of the colon length induced by AOM/DSS (8.64 ± 0.34 cm, *P* < 0.05) as shown in Figure 6F. RT-qPCR results showed that only LATS2 mRNA was abrogated in CAC model. And the decrease of LATS2 mRNA level was reversed by pedunculoside (Figure 7A). Immunohistochemical images for APC showed the strong positive in the cytoplasm, very weak positive in the CAC group, and a relatively stronger positive in the pedunculoside group (Figure 7B); On the contrary, immunohistochemical results for YAP showed that the YAP level was very low in normal colon segment, while a significantly enhanced expression of YAP was observed in nucleus for the CAC group.

On the contrary, p-YAP and APC protein expression were both decreased in abnormal dysplasia part compared with positive cytoplasm expression in normal mucosa (Figure 7B). Moreover, the miRNA levels for the pedunculoside group were also evaluated. To be interest, although miR-223-3p and miR-31-5p were up-regulated in CAC model group, pedunculoside specifically down-regulated miR-31-5p level, without affecting miR-223-3p expression (Figure 7C). Subsequently, the protein levels of APC and factors in the Hippo pathway were examined by western blot analysis (Figure 7D). The results showed that the down-stream genes of miR-31-5p and miR-223-3p, including APC and p-YAP, were all decreased in CAC group, which were also rescued by pedunculoside treatment.

## Discussion

Although miRNAs have been linked to the pathogenesis of CAC, the miRNA-based drug screening for CAC are all still out there to be tapped into. More and more evidence implied that the miRNA expression varies dynamically during the oncogenesis of CAC, which means there are chances of preventing CAC by means of reversing the dysregulated miRNAs at different stages of CAC.

In this study, we found in the CAC group, miR-31-5p level was ∼100 times as high as in the normal group, and consistent results were also obtained in Caco2 cells treated with LPS of 72 h. In addition to that miR-31 was overexpressed in intestinal epithelium cells (IECs) of UC and colon cancer (Jacob et al., 2017; Kim et al., 2017; Gwiggner et al., 2018), miR-31 was also involved in the tumorigenesis and intestinal regeneration driven by intestinal stem cells (ISCs) (Gwiggner et al., 2018). Generally, after injury caused by inflammation or radiation, miR-31 was overexpressed in response to stimuli so as to mitigate the damage by driving both IECs and ISCs proliferation, and protects them against apoptosis (Kim et al., 2017; Tian et al., 2017) rapidly. In this process, YAP was an important gene promoting IECs and ISCs proliferation (Tian et al., 2017). However, so far the exact relationship between miR-31 overexpression and YAP activation in CAC is elusive. In this paper, we found that miR-31-5p targets LATS2, which is the up-stream gene of YAP in the Hippo pathway. LATS2 modulates the phosphorylation of YAP. After phosphorylation by LATS2, YAP can be either degraded, or captured and thereby detained by protein 14-3-3 in the cytoplasm, resulting in the decrease of YAP in the nucleus (Hong et al., 2016). Subsequently, decreased YAP gene in nucleus would suppress both IEC regeneration and intestinal tumorigenesis. In the case of CAC, with the increase of miR-31-5p, the LATS2 level is attenuated, causing the decrease of YAP phosphorylation. And the unphosphorylated YAP would not be degraded or detained in the cytoplasm, which means more YAP would be transferred into the nucleus to trigger both the rapid regeneration and tumorigenesis in intestine.

Adenomatous polyposis coli functions as a colorectal tumor repressor, and is highly mutated in CRC. In the early event of CRC, APC mutation or inactivation can be observed uniquely, and dysregulation of APC gene activates Wnt signaling pathway and cause abnormally of cell adhesion, cell migration, proliferation, differentiation, and other cell functions (Zhang and Shay, 2017). MiR-223-3p was another miRNA that was up-regulated in the CAC model group. To be noted, it was also overexpressed in IBD (Kim et al., 2016; Wang et al., 2016), and was deemed as a biomarker for stage II and III colon cancer (Jacob et al., 2017). In this paper, we identified the APC mRNA as the direct target for miR-223-3p in Caco2 cells for the first time. Overexpressed miR-223-3p in precancerous stage of CAC would thus decrease APC level, contributing to intestinal tumorigenesis.

**FIGURE 8:**
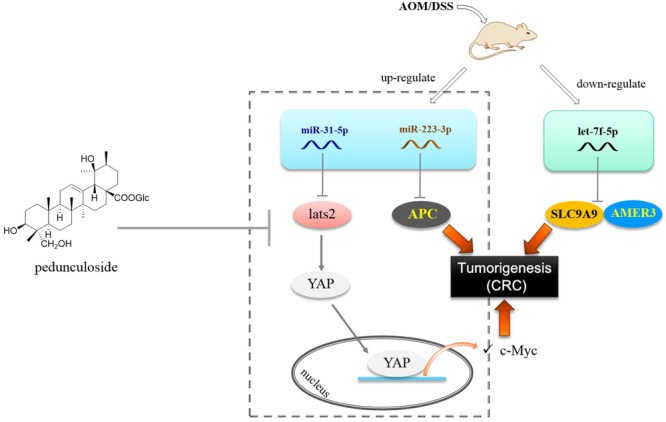
Postulated scheme of pedunculoside preventing AOM/DSS-induced CAC via abolishing the effect of miR-31-5p and miR-223-3p.

Let-7f-5p was found to be decreased in the CAC model, as opposed to miR-223-3p and miR-31-5p that were up-regulated. In this paper, we substantiated that AMER3 and SLC9A9 were two direct targets for let-7f-5p. Amer3 is an activator of Wnt signaling, in contrast to Amer1 and Amer2, Amer3 levels are positively correlated with the level of c-Myc (Brauburger et al., 2014). Furthermore, decreased let-7f-5p would also lead to SLC9A9 increase. SLC9A9 was overexpressed in CRC specimens and up-regulation of SLC9A9 promotes cancer progression and is involved in poor prognosis in CRC (Ueda et al., 2017). Therefore, our finding showed that in the CAC model, the increased miR-31-5p and miR-223-3p, along with the decreased let-7f-5p would trigger and exacerbate CAC via targeting multiple factors, including LATS2 (Hippo pathway), APC, AMER3, and SLC9A9 (Figure 8).

Finally we found that pedunculoside could counteract the effect of miR-31-5p and miR-223-3p overexpression in Caco2 cells. This is interesting for so far we barely know the effect of manipulated miRNA levels in the CAC progression. Pedunculoside belongs to ursane type of triterpenoids. We isolated this effective constituent from *Ilex rotunda* Thunb, which has been used as a Traditional Chinese Medicine to clinically treat abdominal pain and colitis-related diseases over north part of China with the name of “Jiubiying” that literally means “help when needed.” The *in vivo* CAC preventive experiment showed that pedunculoside suppressed the AOM/DSS-elicited intestinal injury, and prevented tumorigenesis via abolishing the effect of both miR-31-5p and miR-223-3p on Hippo pathway and APC (Figure 8). All these outcomes suggested that miRNAs possess the great potential of being new therapeutic targets in diseases like CAC whose pathogenesis involves miRNA aberrant expression. Furthermore, our results also revealed the role of post-transcriptional gene regulation in CAC.

## Author Contributions

GC, NL, and YL designed the manuscript, YF conducted most of the experiments. XL, ZJ, BB, LZ, and YH analyzed the data and wrote the manuscript. All authors critically reviewed and approved the final version of the manuscript.

## Conflict of Interest Statement

The authors declare that the research was conducted in the absence of any commercial or financial relationships that could be construed as a potential conflict of interest.
