# Supplementary material for: Post-transcriptional Gene Regulation in Colitis Associated Cancer
Source: Front Genet. 2019 Jun 19;10:585. doi: 10.3389/fgene.2019.00585 (PMC6593052; doi:10.3389/fgene.2019.00585)
Supplement: Supplementary file 1 [file Table_1.DOCX]

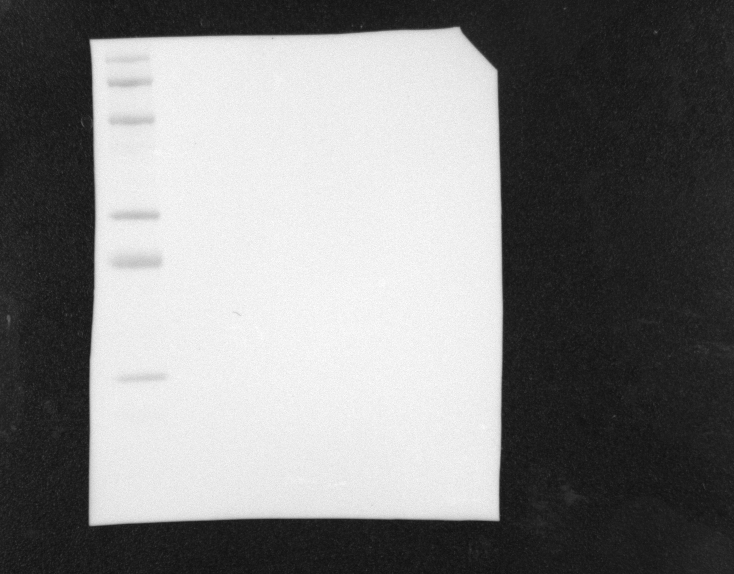


**A**


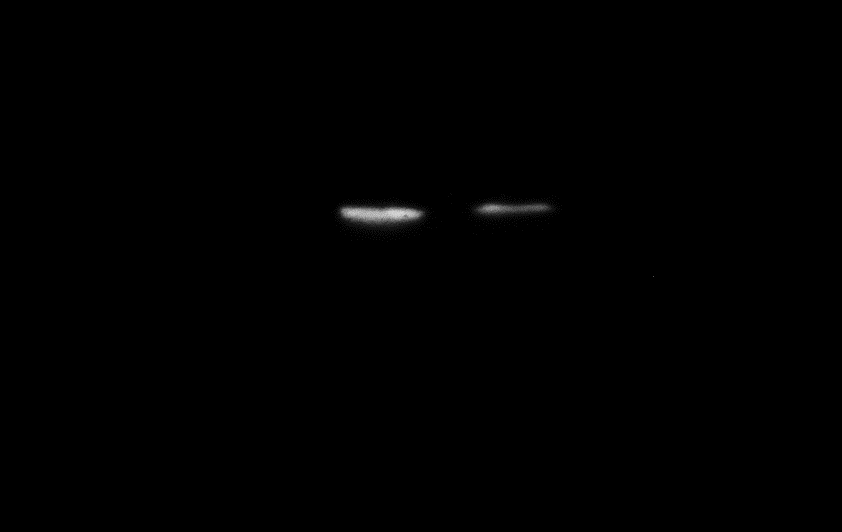


**B**


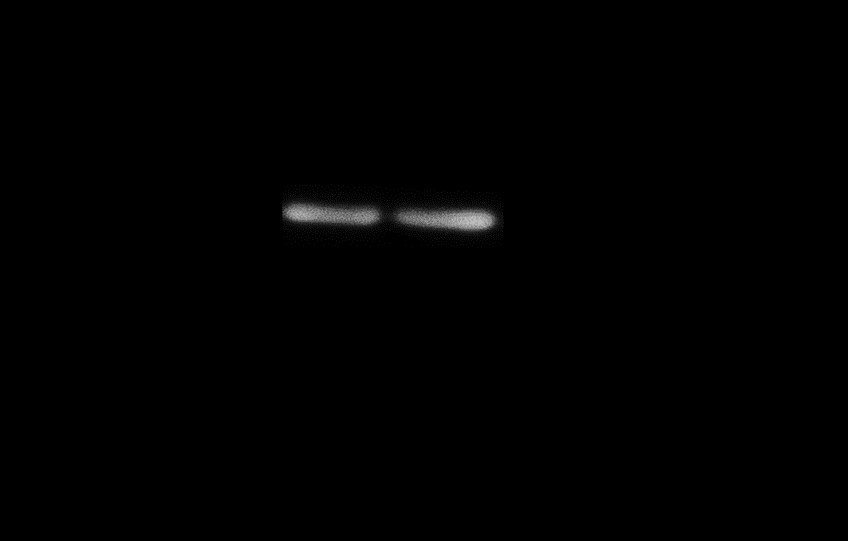


**C**

Figure S1. Gel for figure 2. A: Whole gel. B: APC. C: GAPDH.


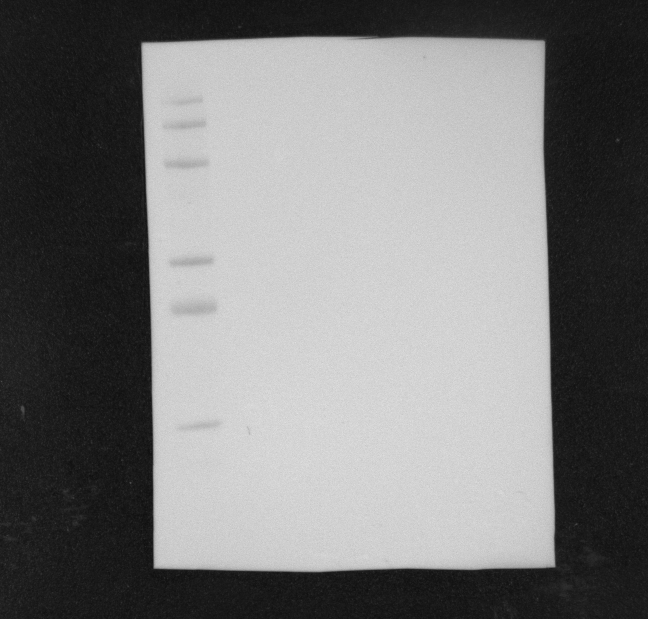


**A**


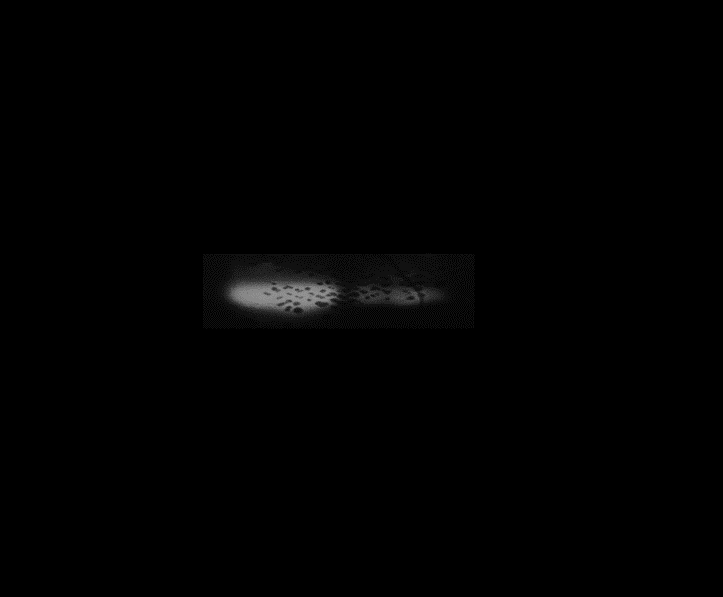


**B**


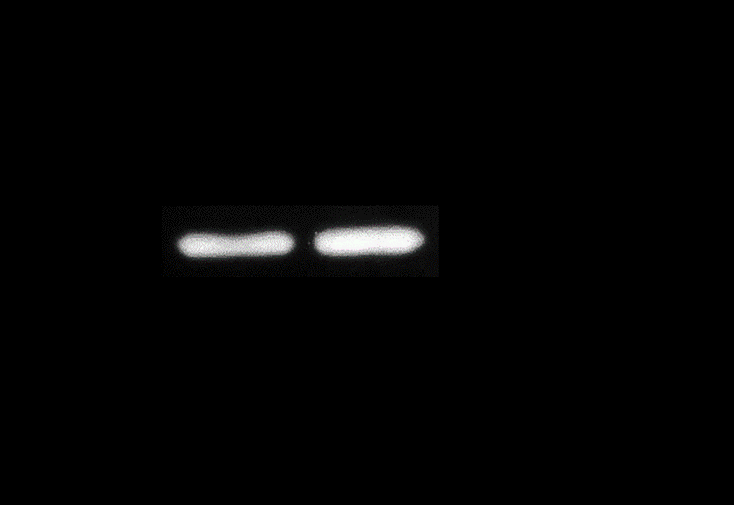


**C**

Figure S2. Gel for figure 3. A: Whole gel. B: AMER3 C: GAPDH.


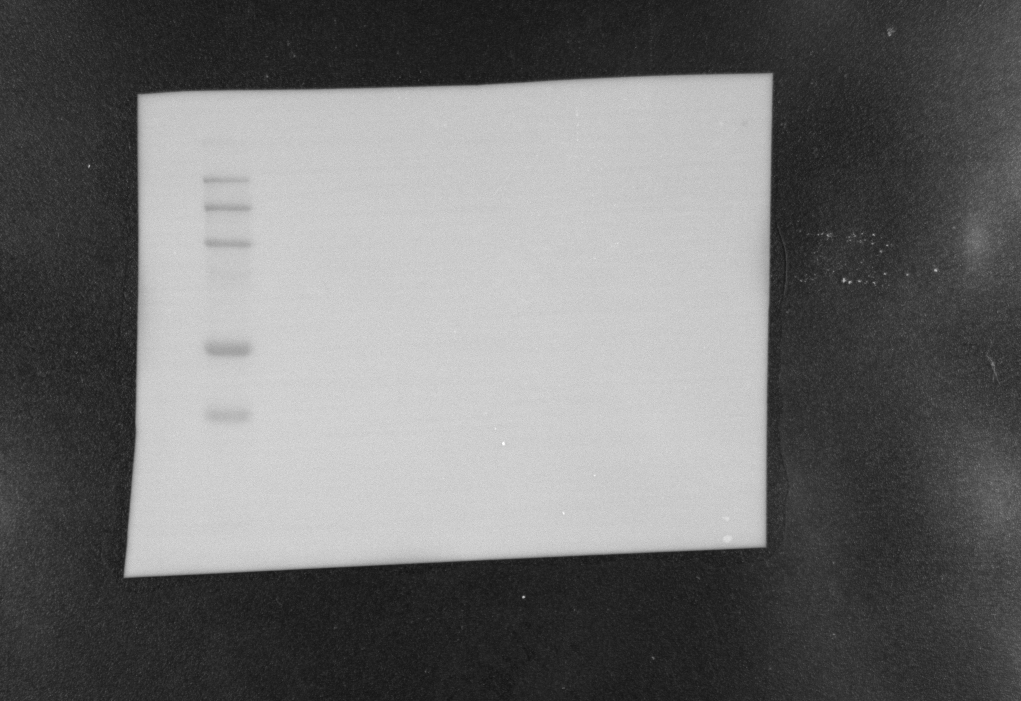


**A**


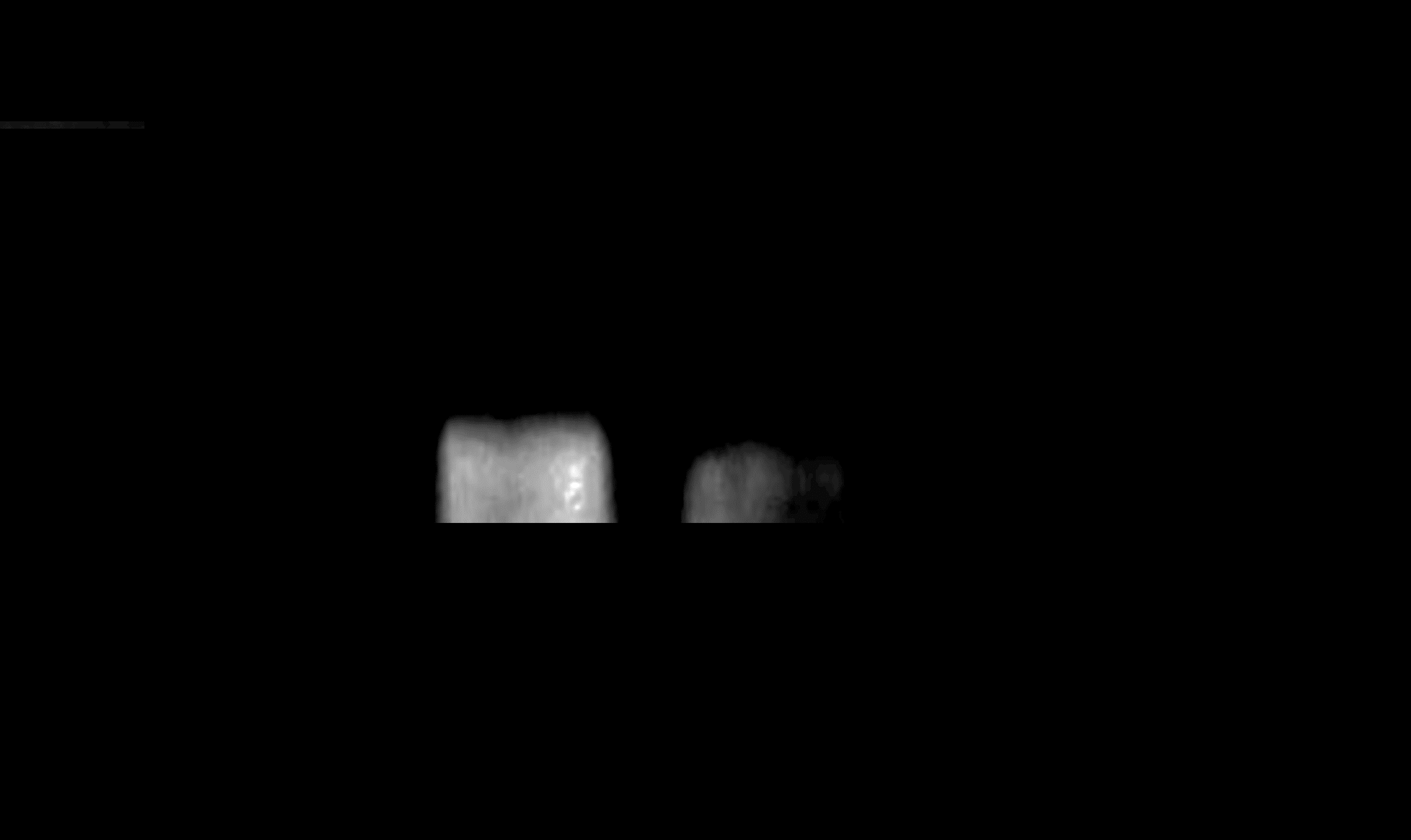


**B**


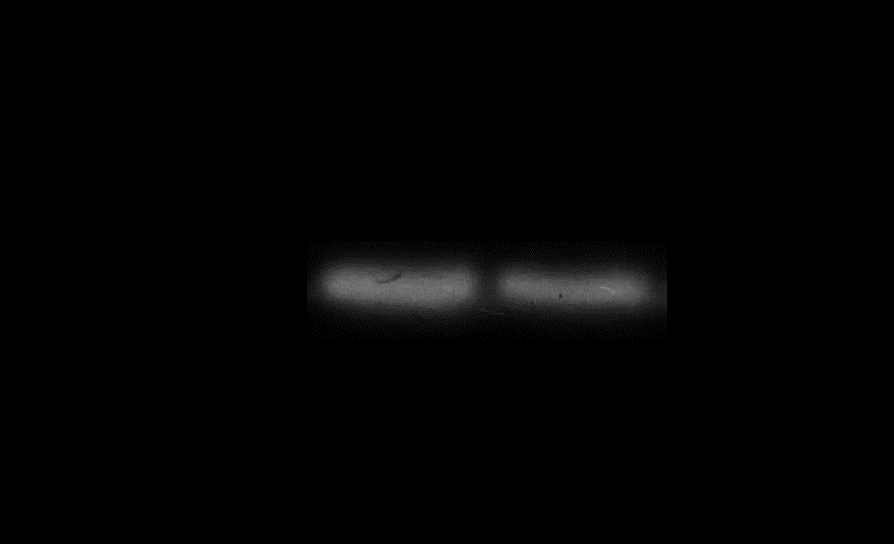


**C**

Figure S3. Gel for figure 3. A: Whole gel. B: SLC9A9 C: GAPDH.


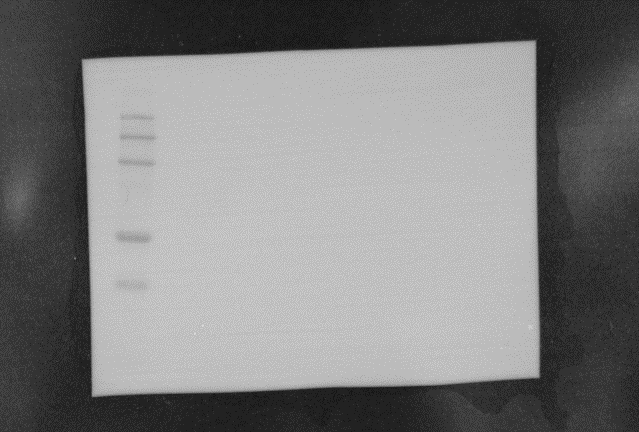

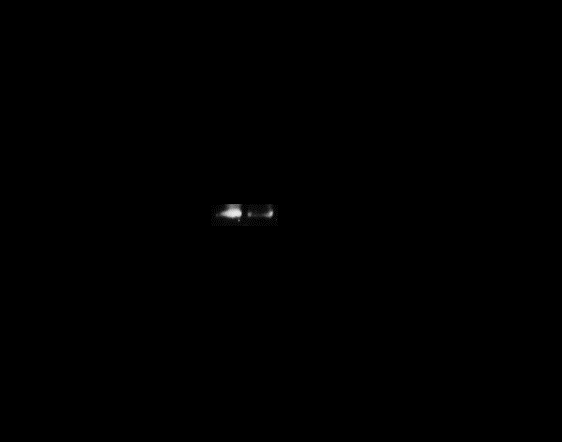

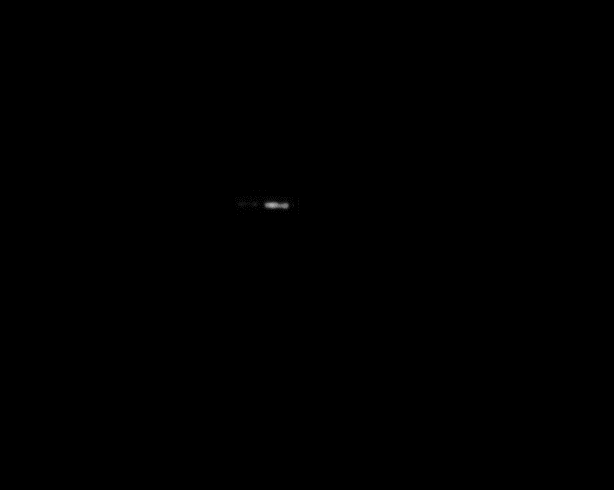

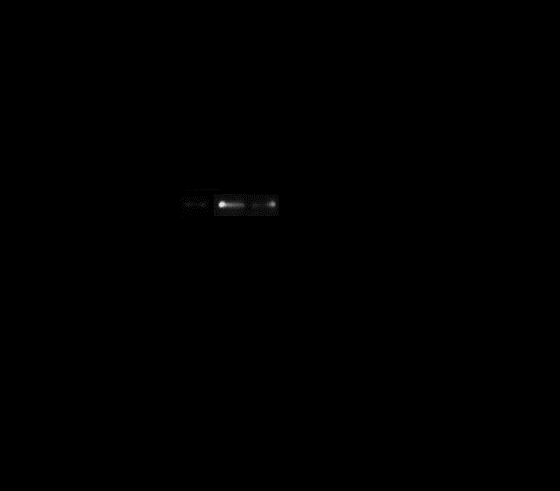


**A**

**C**

**D**

**B**


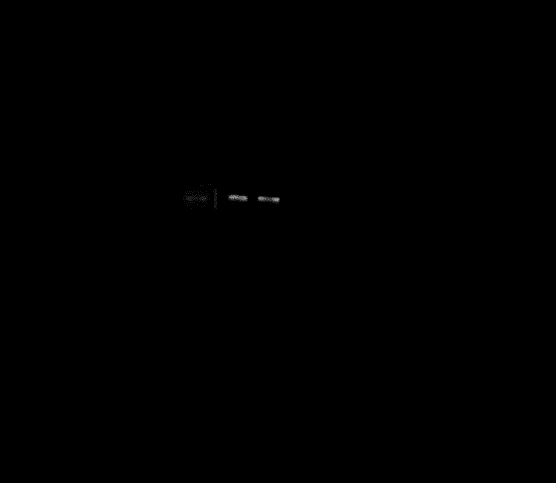

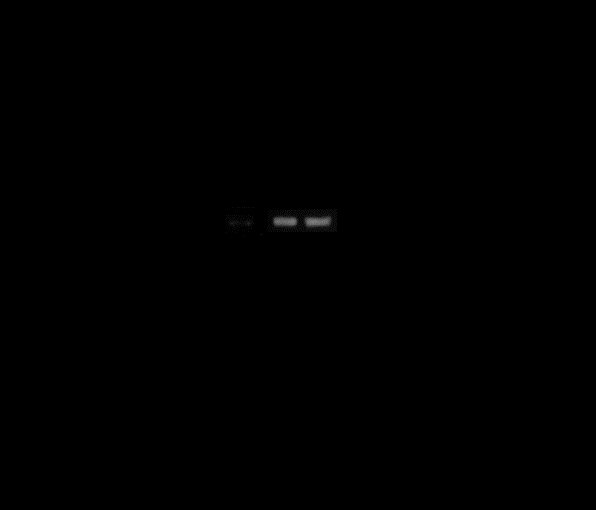


**F**

**E**


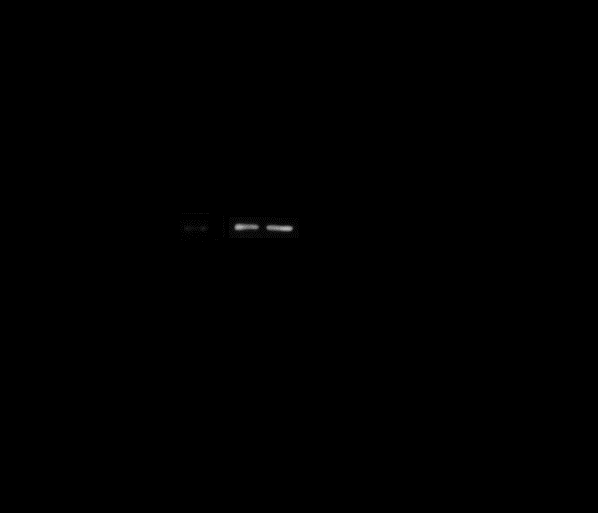

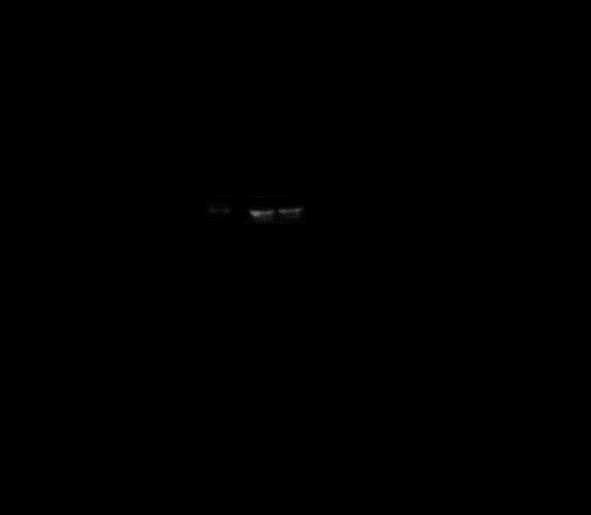


**H**

**G**

Figure S4. Gel for figure 4. A: Whole gel. B: LATS2. C: YAP/TAZ. D: P-YAP. E: LATS1. F: MST1. G: MOB1. H: β-actin


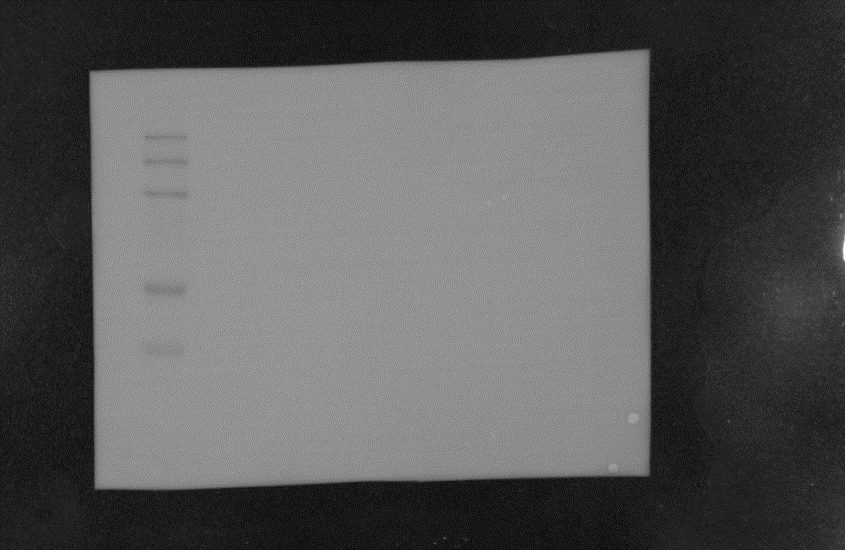


**A**


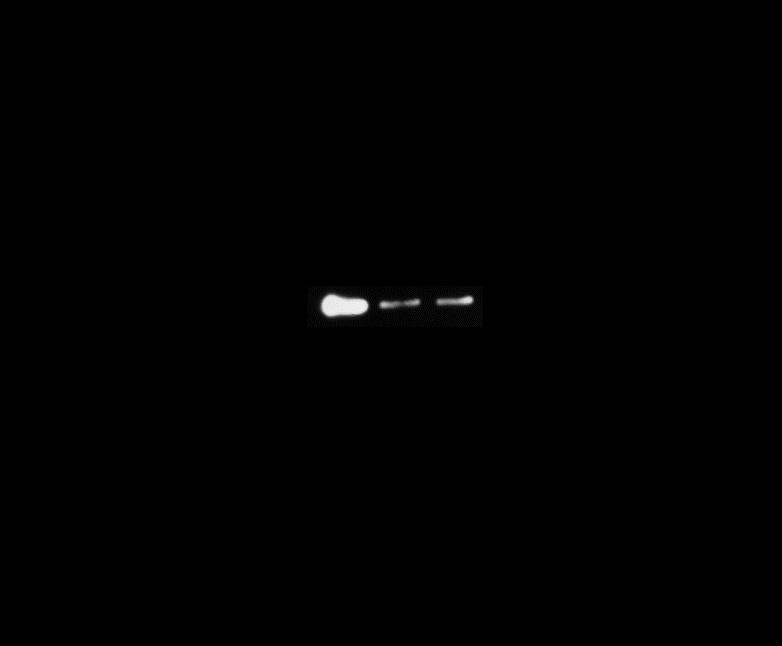


**B**


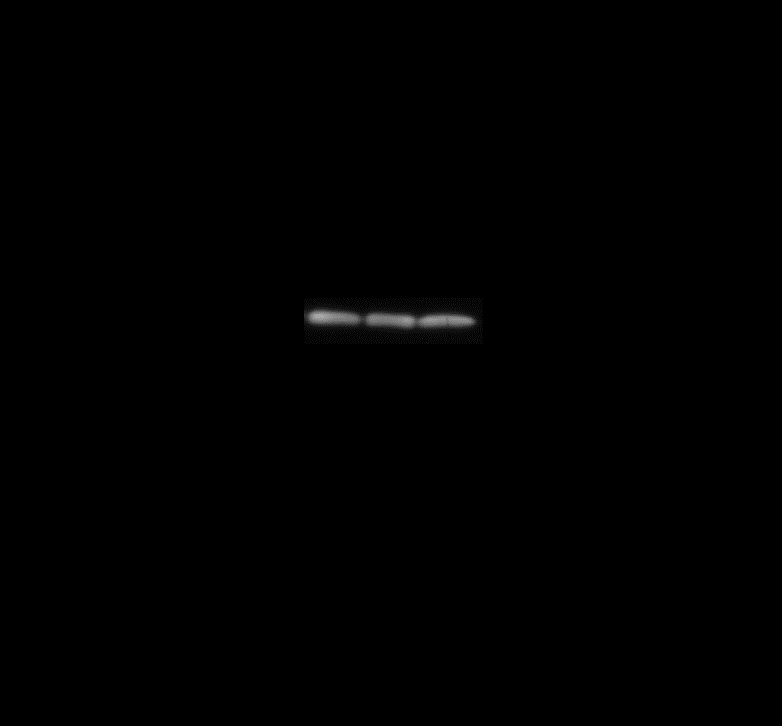


**C**

Figure S5. Gel for figure 5. A: Whole gel. B: LATS2. C: GAPDH.


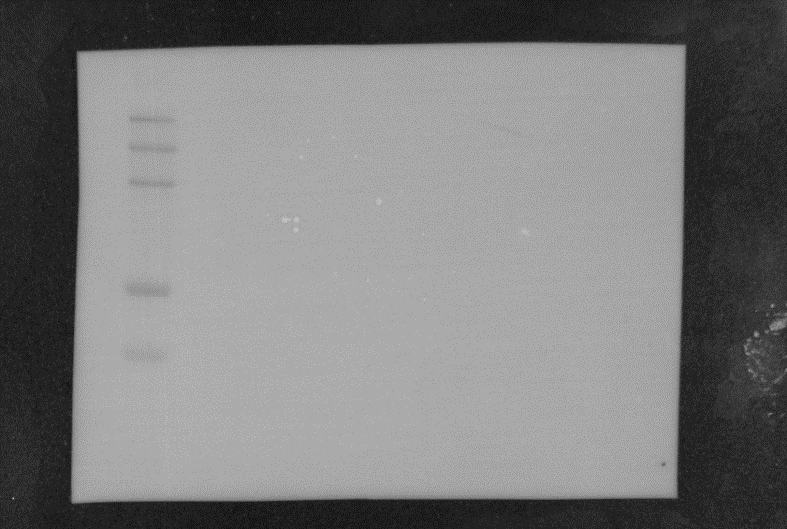


**A**


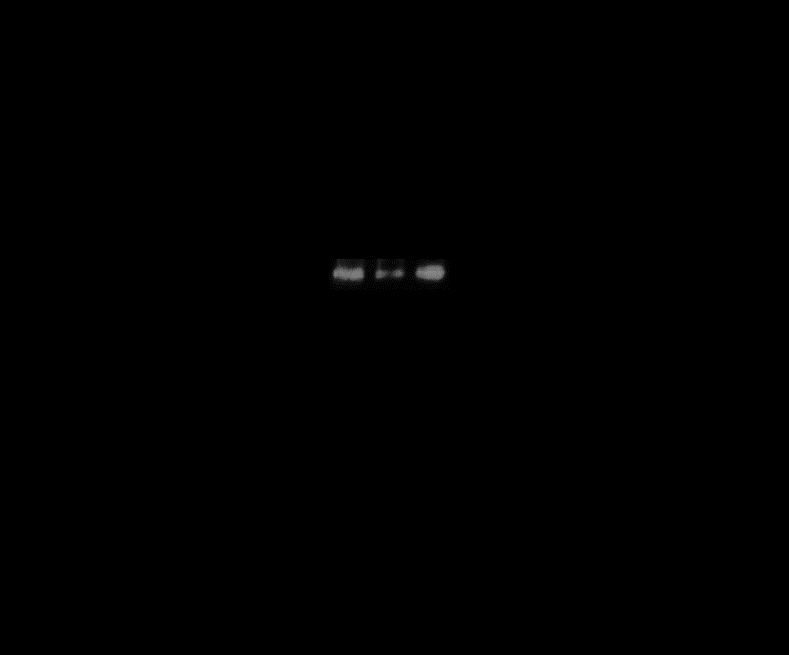


**B**


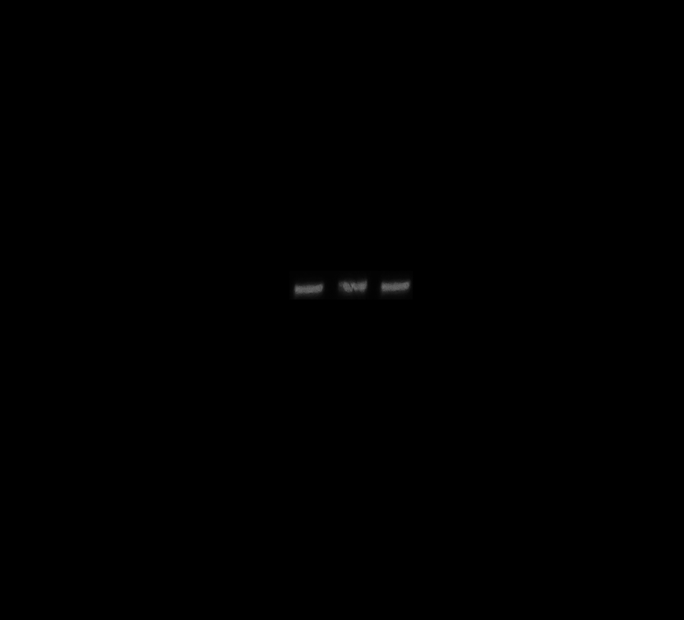


**C**

Figure S6. Gel for figure 5. A: Whole gel. B: APC. C: GAPDH.


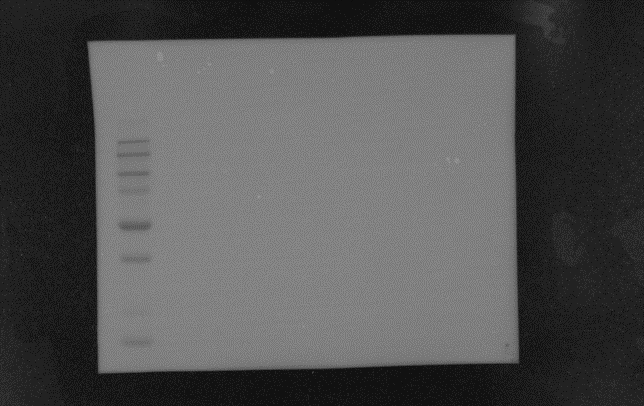


**B**

**A**


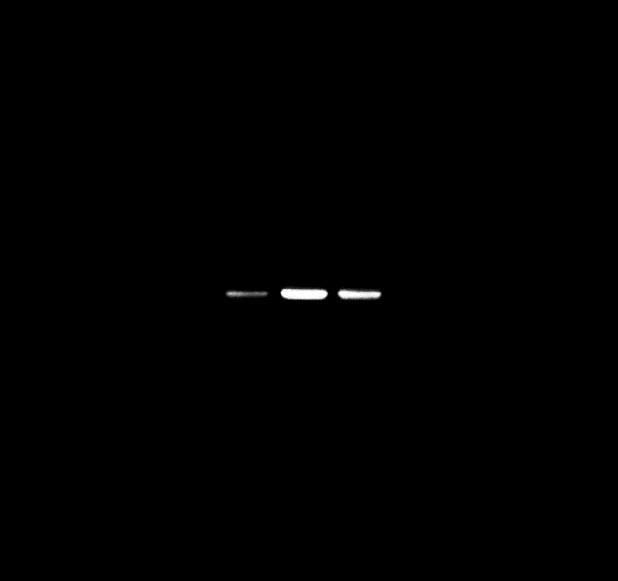

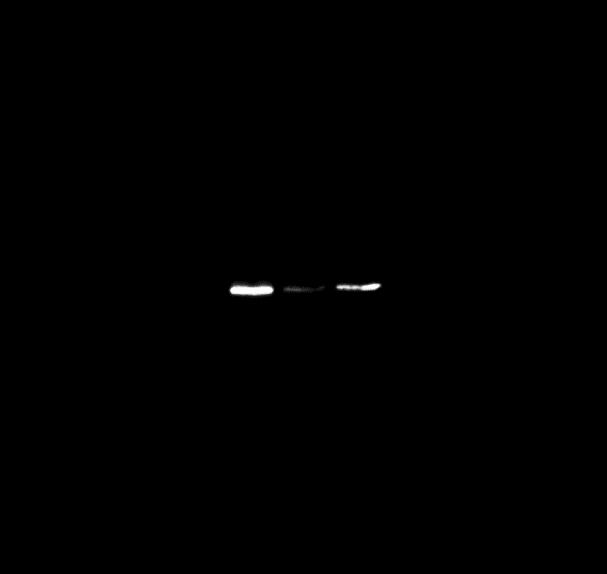


**C**

**B**


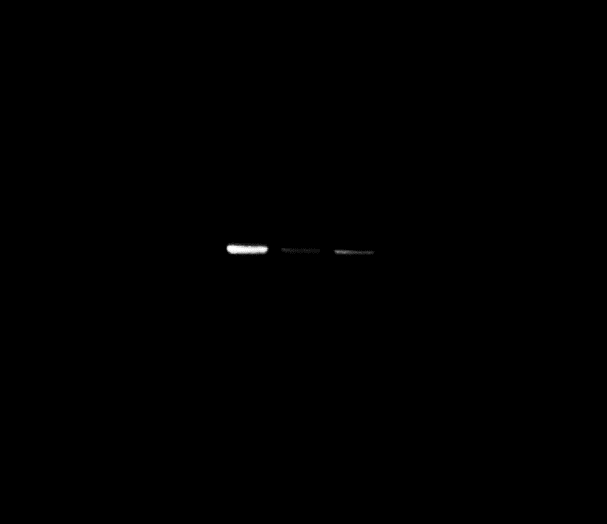

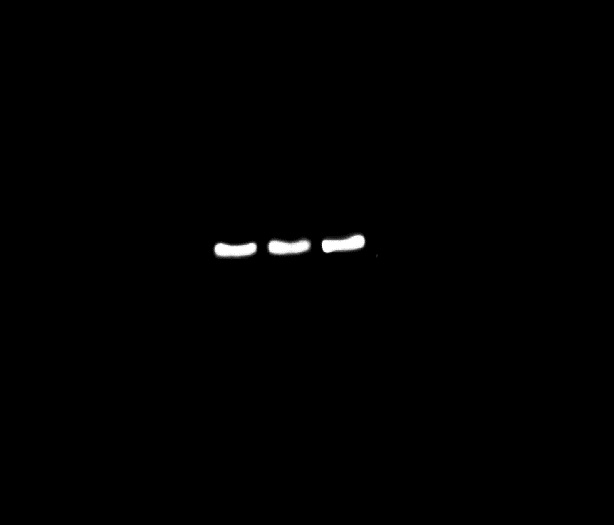


**E**

**D**

Figure S7. Gel for figure 7. A: Whole gel. B: YAP/TAZ. C: P-LATS. D: APC. E: GAPDH.

**Materials and Methods**

*General Experiment*

NMR spectra were measured on Bruker ARX-400 spectrometer, with TMS as an internal standard. Silica gel (200-300 mesh) for chromatography was produced by Qingdao Ocean Chemical Group Co. of China. Pyridine-*d*_5_ was obtained from Sigma-Aldrich Company (St. Louis, MO, USA). All the chromatographic and analytical grade reagents were obtained from Tianjin DaMao Chemical Company (Tianjin, China).

*Isolation for pedunculoside*

The EtOAc extract (40g) of *Ilex rotunda* was subjected to silica gel column (PE-EtOAc, 100:0-0:100). Then similar fractions were collected together on the basis of similarity in R*_f_* values and afforded 9 fractions (Fr. 1-Fr. 9). Fr. 9 (10.3 g) were purified using repeated crystallization from CH_2_Cl_2_-MeOH to respectively afford compound**17** (1.0 g).

*Identification for pedunculoside*

Colorless needle crystal (MeOH). ^1^H-NMR (400 MHz, Pyridine-*d*_5_): *δ* 6.30 (1H, d, *J* = 7.6 Hz, Glc-H-1), 5.59 (1H, br. s, H-12), 1.66 (3H, s, 29-CH_3_), 1.42 (3H, s, 24-CH_3_), 1.24 (3H, s, 26-CH_3_), 1.09 (3H, d, *J* = 6.5 Hz, 30-CH_3_), 1.08 (3H, s, 27-CH_3_), 1.05 (3H, s, 25-CH_3_). ^13^C-NMR (100 MHz , Pyridine-*d*_5_): *δ* 39.6 (C-1), 27.4 (C-2), 73.8 (C-3), 43.3 (C-4), 49.5 (C-5), 19.3 (C-6), 33.6 (C-7), 41.2 (C-8), 48.0 (C-9), 37.9 (C-10), 24.7 (C-11), 129.7 (C-12), 139.6 (C-13), 42.7 (C-14), 29.7 (C-15), 26.5 (C-16), 49.1 (C-17), 55.0 (C-18), 73.6 (C-19), 42.9 (C-20), 27.1 (C-21), 38.3 (C-22), 67.5 (C-23), 12.7 (C-24), 17.6 (C-25), 16.6 (C-26), 24.7 (C-27), 177.3 (C-28), 27.2 (C-29), 16.3 (C-30), 95.8 (Glc-1), 74.0 (Glc-2), 78.6 (Glc-3), 71.1 (Glc-4), 78.3 (Glc-5), 62.4 (Glc-6).
